# Formulated hydroxy fatty acids from fruit pomaces reduce apple scab development caused by *Venturia inaequalis* through a dual mode of action

**DOI:** 10.3389/fpls.2023.1322638

**Published:** 2024-01-08

**Authors:** Matthieu Gaucher, Anthony Juillard, Bao-Huynh Nguyen, Noémie Viller, Cédric Ernenwein, Didier Marion, Marie-Noëlle Brisset, Bénédicte Bakan

**Affiliations:** ^1^ Univ Angers, Institut Agro, INRAE, IRHS, SFR QUASAV, Angers, France; ^2^ INRAE, Biopolymers Interactions Assemblies, Nantes, France SDP Rovensa Company, Laon, France; ^3^ SDP Rovensa Company, Laon, France

**Keywords:** hydroxy fatty acids, cutin, tomato, apple, pomaces, defense, protection, apple scab

## Abstract

The outermost hydrophobic layer of plants, i.e. the cuticle, is mainly composed of cutin, a polyester of hydroxy fatty acids with reported eliciting and/or antimicrobial activities for some of them. By-products of the fruit processing industry (fruit pomaces), often strongly enriched in cuticular material, are therefore a potential source of bioactive compounds for crop protection against pathogen attack. We investigated the utilization of tomato and apple pomaces in the development of a cutin-based biocontrol solution against apple scab, a major apple disease caused by *Venturia inaequalis*. Several cutin monomer extracts obtained through different strategies of depolymerization and purification were first compared for their ability to induce a targeted set of defense genes in apple seedlings after foliar application. After a step of formulation, some extracts were chosen for further investigation *in planta* and *in vitro*. Our results show that formulated cutin monomers could trigger a significant transcriptome reprogramming in apple plants and exhibit an antifungal effect on *V. inaequalis*. Cutin monomers-treated apple seedlings were significantly protected against infection by the apple scab agent. Altogether, our findings suggest that water-dispersed cutin monomers extracted from pomaces are potential new bio-based solutions for the control of apple scab.

## Introduction

The development of alternative pest control strategies is essential to meet the expectations of consumers on the reduction of pesticides and the increasing ban of controversial synthetic products by the European Union (European Union, 2009). In that respect, finding new environmentally friendly bioactive candidates for pest control is urgently needed.

The plant cuticle covering every aerial organ has interesting properties. The polymeric scaffold of the plant cuticle, i.e. the cutin, is a polyester of C_16_ and/or C_18_ hydroxy fatty acids including waxes and polysaccharides (Reynoud et al., 2021). Besides its role as a physical barrier, the cuticle is a source of Damage Associated Molecular Patterns (DAMPs) able to induce plant immunity (Hou et al., 2019). Exogenous application of solvent-solubilized cutin monomers could confer resistance to *Erysiphe graminis* and *Magnaporthe grisea* in barley and rice respectively, without exhibiting fungicidal activity (Schweizer et al., 1994) and stimulate various defense mechanisms such as the production of ethylene and H_2_O_2_ or the overexpression of PR (pathogenesis-related) genes (Schweizer et al., 1996; Fauth et al., 1998; Kauss et al., 1999; Park et al., 2008; Buxdorf et al., 2014). Recently, the bactericidal activity of mixtures of cutin oligomers and monomers towards *Escherichia coli* and *Staphylococcus aureus* has been also described (Escórcio et al., 2022).

Cutin monomers appear therefore to be good candidates in a strategy to reduce the use of conventional pesticides through a potential dual mode of action. One of the key issues is i) to get a sufficient and stable source of cutin monomers and ii) to be able to disperse these lipids in water for their subsequent agronomic use. Interestingly, the fruit processing industry generates high amounts of cutin-rich by-products representing 3-5% (w:w) of the processed biomass (Benítez et al., 2018). Tomato fruit is one of the most produced crops and concentrates a high amount of cutin (70% w/w) (Osman et al., 1999), whose monomer composition is strikingly homogenous with about 80% by weight consisting of 9(10),16-dihydroxy hexadecanoic acid (Benítez et al., 2018; Marc et al., 2021; Escórcio et al., 2022). Apple pomaces also contain a substantial amount of cutin (14% w/w) (Mellinas et al., 2022), and could therefore constitute another industrial source of hydroxy fatty acids. Besides, tomato and apple cutin monomer compositions are different (Kosma et al., 2010; Lara et al., 2014; Leide et al., 2018) which could impact their biological activity (Schweizer et al., 1996; Escórcio et al., 2022). In addition, using lysine and choline as counter-ions, these hydroxy fatty acids can spontaneously disperse in water as stable monolayer vesicles (Fameau et al., 2013).

The use of such water-dispersed cutin monomers from industrial pomaces for controlling plant diseases has never been investigated. For such studies, the choice of apple (*Malus domestica*) is particularly justified since this major fruit tree species needs extensive pesticide treatments, especially for the control of apple scab (caused by *Venturia inaequalis*) which requires more than 12 fungicide treatments each season (Chatzidimopoulos et al., 2020). *V. inaequalis* is a hemibiotrophic fungus that uses an uncommon strategy, by forming a parasitic mycelium in the subcuticular space of leaves and fruits without host cell penetration (MacHardy, 1996).

The present work presents the first steps in the development of an eco-friendly agricultural product capable of controlling apple scab and based on cutin monomers-enriched extracts. Different processes of extraction and purification from tomato pomaces and one process of extraction from apple pomaces were compared for the ability of the resulting extracts to induce a targeted set of defense genes following foliar application on apple seedlings. An apple transcriptome analysis after treatment as well as *in vitro* antifungal tests against *V. inaequalis* were carried out to further investigate the mode of action of selected and formulated tomato extracts. This finally led us to select a high-performance formulation of cutin monomers isolated from peels of tomato pomaces able to protect, in a preventive way, young apple plants from apple scab in controlled conditions.

## Materials and methods

### Production of hydroxy fatty acid extracts

The hydroxy fatty acid-rich fractions (**Table 1**) were extracted from industrial tomato (extracts E1 to E6) and apple pomaces (extract E7) provided by the “Conserveries de Bergerac” (UNIPROLEDI, Bergerac, France). The peels were isolated from tomato pomaces, dried, and grounded as previously described (Marc et al., 2021). To concentrate the cutin fraction, apple pomaces were dispersed in hot tap water (50°C) for 1 h. Then, the mixture was filtered on 0.2 mm stainless steel sieves and the solid retentate was thoroughly washed with tap water. The water excess was squeezed out by a homemade press and dried at room temperature. Except for extracts E3 and E4, grounded peels (1 kg) were dewaxed by acetone: ethanol 1:1 (v: v) in a Soxhlet extractor and then dried in a fume hood. Cutin depolymerization was conducted at room temperature for 24 h in 5% KOH (w:v) either in ethanol 95% (Marc et al., 2021) for extracts E1, E3, E5, E6 and E7 and or in 5% (w:v) NaOH in water (Cigognini et al., 2015), for extracts E2 and E4. Extracts were filtered on a Buchner funnel, and about 90% of the filtrate was evaporated under vacuum and replaced by water. Hydroxy fatty acids were precipitated by adjusting the pH to 3.5 with concentrated HCl. The hydroxy fatty acid precipitate was extensively washed with water, and finally freeze-dried.

**Table 1:** Main characteristics of the cutin monomer extracts.

Extracts	Source	Abbreviation	Concentrations used (g/l)	Batch	Hydrolysis solvent	Waxes	Discolored	Formulated
Extract 1	Tomato	E1	0.5	1	EtOH			
Extract 2	Tomato	E2	0.5	1	H_2_O			
Extract 3	Tomato	E3	0.5	1	EtOH	x		
Extract 4	Tomato	E4	0.5	1	H_2_O	x		
Extract 5	Tomato	E5	0.5	2	EtOH		x	
Extract 6	Tomato	E6	0.5	2	EtOH			
Extract 7	Apple	E7	–	1	EtOH			
Extract 1	Tomato	E1F	0.25 to 2	1	EtOH			x
Extract 3	Tomato	E3F	0.25 to 2	1	EtOH	x		x
Extract 7	Apple	E7F	1.5	1	EtOH			x

For fraction E5, tomato cutin monomers were further purified to remove the co-extracted phenolic compounds by using an adsorption chromatography process (Kaluzny et al., 1985). The crude cutin monomers extract (10 g) was applied in chloroform-2-propanol 2:1 (v:v) onto an aminopropyl-silica gel column (Macherey Nagel) (100 g). After washing the column with this solvent, the hydroxy fatty acids were eluted with chloroform. After solvent evaporation under vacuum, the lipid composition was determined by GC-FID (Marc et al., 2021). Total phenolic (TP) content was determined by a colorimetric Folin-Ciocalteu (Singleton et al., 1999) using gallic acid (Sigma) and catechin (Sigma) for the tomato and apple phenolic calibration curves, respectively. TP was expressed as mg of gallic acid equivalents or catechin equivalents per g of cutin monomers extracts.

Cutin monomer extracts were analyzed by attenuated total reflectance Fourier-transform infrared spectroscopy (ATR-FTIR). Spectra (50 scans) were acquired in the 4000-700 cm^-1^ range at a resolution of 2 cm^-1^ on a Magna-IR 550 spectrometer using a single reflection accessory with a 45° angle of light incidence on a diamond crystal. The instrument was continuously purged with dry air.

Cutin monomers were either dispersed in reverse osmosis water at a concentration of 0.5 g/l, pH 8.5 (fractions E1 to E6) or formulated according to the SDP Rovensa (Laon, France) formulation using a bio-based lysine counterion, as previously described (Fameau et al., 2013), and containing 15% (w:v) of cutin monomers for stock solutions (pH 8.5). These stock solutions were then diluted in reverse osmosis water to reach concentrations ranging from 0.25 to 2 g/l of cutin monomers (fractions E1F, E3F, E7F). The co-formulants alone were diluted to concentrations similar to the working concentrations of extracts (formulation blank FB). The absence of pesticide residues was checked for all the fractions used (Eurofins analysis, France).

### Plant material and treatments

All experiments were performed on apple seedlings (4–6 leaves) from open-pollinated cv. Golden Delicious. Plants were grown in individual pots (8 x 5.5 x 7 cm) under greenhouse conditions (natural photoperiod supplemented with artificial light if needed, 17°C night and 20–23°C day). Crude or formulated cutin monomer extracts (and reverse osmosis water or FB as respective control) were sprayed to runoff on entire plants with a spray gun Aeryo-1.4 (Deltalyo, Mably, France). For all assays, plants received two treatments with extracts (or controls) four days apart, and inoculation of *V. inaequalis* or sampling for defense analysis (RT-qPCR and RNAseq) occurred 2 or 3 days after the second treatment respectively (**Supplementary Figure S1**). The youngest expanded leaf on the day of the second treatment was labeled for sampling or scab symptom tracking.

### Apple defense induction

Each sample (biological replicate) corresponds to a bulk of the youngest expanded leaf from five plants per treatment (extracts or control). Hundred mg of leaf tissues were ground (Retsch, Haan, Germany) and total RNA was extracted with the Macherey-Nagel Nucleospin RNA Plant Kit (Macherey-Nagel, Düren, Germany) and by adding 2% PVP-40 in the extraction buffer.

#### RT-qPCR

Reverse transcription was performed as described by Promega (M-MLV) and the complete absence of gDNA checked by polymerase chain reaction (PCR) using EF-α (elongation factor α) primers flanking an intron, as described in Gilliland et al. (1990). Quantitative real-time PCR measurements were performed using a patented set of primers for 29 defense genes and 3 reference genes (Brisset and Dugé de Bernonville, 2011). PCR mixes, data acquisition and calculation of relative changes in defense gene expression (log_2_ ratios) were carried out as described in Gaucher et al. (2022). The value of water- or FB-treated samples was used for the calculation of the log_2_ ratio of each defense gene. Data were obtained from two biological replicates per treatment (extracts or control) and per experiment in two to three independent experiments (n = 4 or 6).

#### RNAseq

Libraries were generated using the Illumina mRNA Stranded protocol and sequenced with the Illumina NovaSeq 6000 S4 PE100 reads technology (Génome Québec, Canada). The sequenced reads (ranging from 23 to 44.6 million read pairs per sample) were mapped on the reference transcriptional units from GDDH13 v1.1 using Salmon software (Patro et al., 2017). Mapping rates ranged from 86.8 to 89.2%. The original sequencing datasets have been deposited in the Gene Expression Omnibus (GEO) with the accession number GSE245564. Identification of differentially expressed genes (DEGs) was performed with DESeq2, including a Benjamini–Hochberg procedure to control the false discovery rate (FDR) (Love et al., 2014). Gene Ontology (GO) enrichment analyses were run with the topGO R package using the default parameters. Data were obtained from one biological replicate per treatment (extract or control) and per experiment in three independent experiments (n = 3).

### Isolate *of V. inaequalis*, *in vitro* antifungal activity and apple-protection tests

The single-spore isolate 104 (Guillaumès et al., 1995) was multiplied on cellophane sheets overlaid onto potato dextrose agar (PDA, 39 g/l) medium supplemented with yeast extract (3 g/l). After seven days of incubation at 17°C, the cellophane sheets were dried and stored at -20°C until use. Conidia suspensions were extemporaneously prepared by placing cellophane sheets in reverse osmosis sterile water under agitation to obtain the desired suspension of conidia.

For the study of *in vitro* antifungal activity, 200 µl of a conidial suspension (1.5 × 10^5^ conidia/ml) were first spread onto a new PDA medium. Twenty days after incubation at 17°C, inoculum plugs (medium+culture, ∅ 9 mm) were cut out using a cork-borer and deposited on a new PDA medium containing the formulated tomato cutin monomer extracts (1.5 g/l), FB, captan (1.87 g/l, positive control) or sterile water (negative control). Diameters of mycelial mats were measured 12 days after incubation at 17°C. Data were obtained from 3 plugs per Petri dish per condition (extract, FB, captan or water) and per experiment in four independent experiments (n = 12).

For protection tests, conidial suspensions (1.5 × 10^5^ conidia/ml) were sprayed on entire plants to runoff with a pressurized hand sprayer. After inoculation, plants were maintained 2 days in high-humidity chambers (darkness, 18°C, 95% relative humidity (RH)) and incubated afterward in controlled conditions (16 h light/8 h dark, constant temperature of 18°C, 70% RH). The percentage of leaf surface exhibiting sporulating lesions was scored 2 and 3 weeks after inoculation on each labeled leaf (having received the treatments) and the one above the labeled leaf (developed after the second treatment). Data were obtained from 30 plants per treatment (extracts or control) and per experiment in three independent experiments (n = 90).

### Statistical analyses

Statistical analyses were performed using RStudio software (R Core Team, 2019). All significant differences (gene expression by RT-qPCR, protective or biocidal effects) were determined by using the nonparametric rank-based statistical tests Wilcoxon-Mann-Whitney and Kruskal-Wallis (P = 0.05). In this last case, Fisher’s Least Significant Difference (LSD) was used as the *post hoc* test for pairwise comparisons (P = 0.05).

## Results

### Extraction of hydroxy fatty acids from tomato and apple pomaces

Cutin monomer extracts were produced from tomato (E1 to E6) and apple (E7) peel-enriched fractions of industrial pomaces through alkaline hydrolysis, either in water or in ethanol (**Table 1**). As tomato fruit cuticles contain low amounts of waxes (0.01 mg.cm^-2^, Kosma et al., 2010) conversely to apple cuticles (up to 1 mg.cm^-2^, Martin and Rose, 2014), we tested whether a preliminary dewaxing step could be avoided for tomato pomaces to simplify the process.

**Figure 1:**
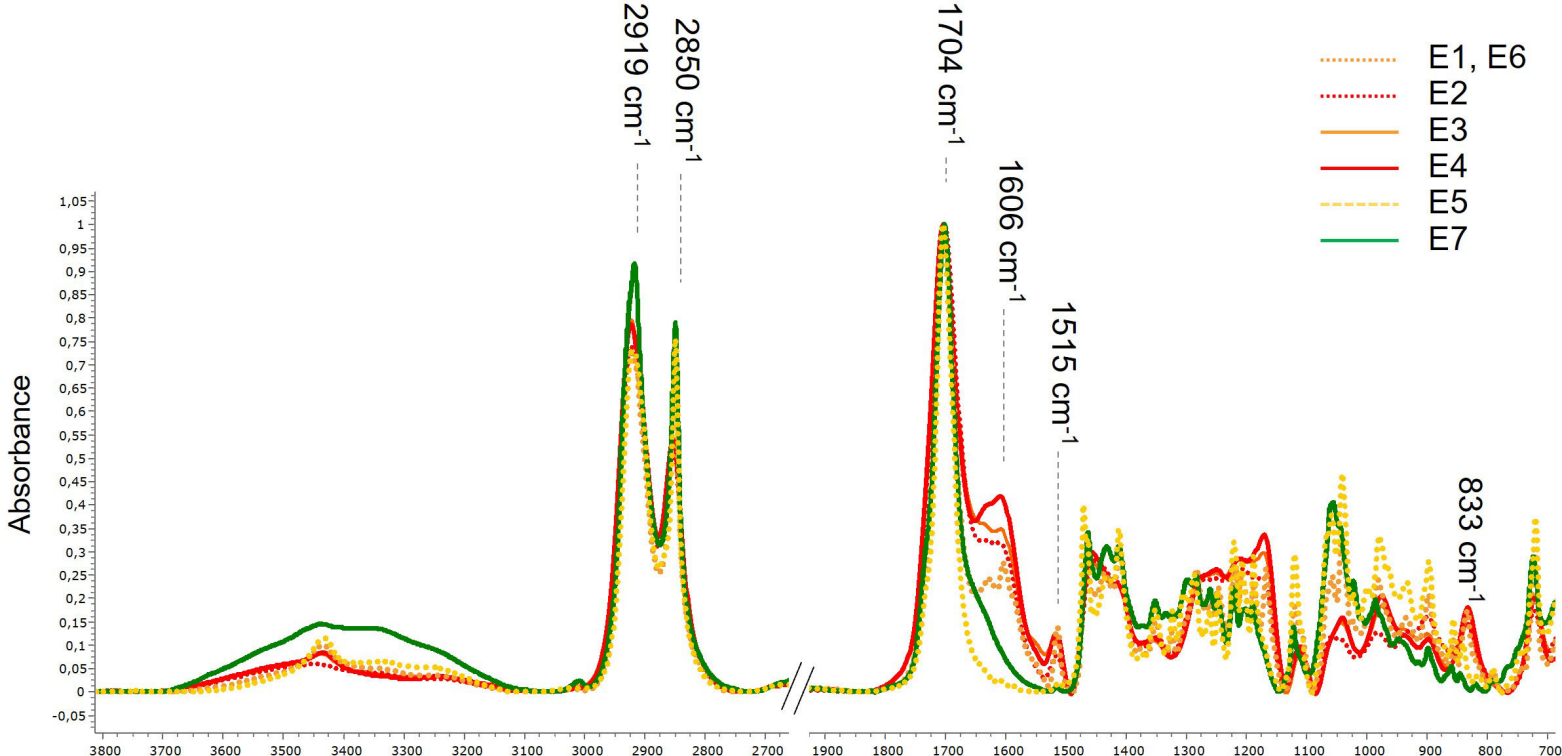
Normalized attenuated total reflectance–Fourier transform infrared spectroscopy (ATR-FTIR) spectra of the cutin monomer fractions.

The maximum yield of hydroxy fatty acids was 55% (w:w) of dried tomato peels and 14% (w:w) of dried apple peels, respectively. The lipid composition of the cutin monomers was assessed (**Table 2**). Both alkaline hydrolysis processes (either in water or in ethanol) of the tomato pomaces, whatever the batch production (**Table 1**), generated cutin monomer extracts highly concentrated in 9(10),16-hydroxy-hexadecanoic acid, in full agreement with the known tomato cutin composition. Minor phenolic compounds were also released by the alkaline hydrolysis of the pomaces as previously described (Perea-Domínguez et al., 2018). The co-extracted phenolics were slightly higher with the water extraction (**Table 2**). In addition, the alkaline hydrolysis in water induced swelling of the extract, making filtration more difficult. The composition of the apple extract is more complex and consists of both C16 and C18 hydroxy fatty acids. These cutin monomers were further characterized by FTIR. The spectra were dominated by intense stretching vibrations of the asymmetric and symmetric methylene chains at 2919 cm^−1^ and 2850 cm^−1^ respectively, and a sharp band at 1704 cm^−1^ assigned to the carbonyl stretch of COOH (**Figure 1**). A broad hydroxyl (3300–3400 cm^−1^) band was also observed. All these features are typical signatures of hydroxy fatty acids (Benítez et al., 2015; Chaudhari and Singhal, 2015; Marc et al., 2021). The FTIR analyses further indicated that, according to our depolymerization process, the extracts were free of polysaccharides known to be present in the plant cuticle of peel fractions (Heredia-Guerrero et al., 2014; Philippe et al., 2020). Furthermore, as the carbonyl band shift from 1730 cm^-1^ to 1704 cm^-1^ upon de-esterification of the cutin polyester (Heredia-Guerrero et al., 2011; Chaudhari and Singhal, 2015; Marc et al., 2021), the 1704 cm^-1^ band further indicated that cutin depolymerization was complete. Minor phenolic compounds were also co-extracted as evidenced by FTIR (specific bands at 1606 cm^-1^, 1515 cm^-1^ and 833 cm^-1^) as well as biochemical analyses (**Table 2**, **Figure 1**). Conversely, the total phenolic content of fractions E5 and E7 was very low. Consequently, we produced from fruit pomaces a set of hydroxy fatty acid fractions including different lipid compositions and contents of co-extracted phenolic compounds. These extracts have been used for plant defense elicitation evaluation.

**Table 2:** Lipid and total phenolic composition of the cutin monomer fractions.

		E1	E2	E3	E4	E5	E6	E7
Lipid composition	hexadecanoic acid	0.2%	1.3%	2.1%	1.2%	0.3%	0.2%	2.4%
	octadecenoid acid	nd	2.2%	0.6%	0.2%	nd	0.2%	2%
	octadecadienoic acid	nd	0.6%	2.7%	1.1%	nd	nd	1.5%
	16-hydroxy-hexadecanoic acid	3.7%	4.3%	3.3%	4.9%	1.4%	3.5%	5.9%
	9(10)-16-hydroxy-hexadecanoic acid	96.1%	91.6%	91.8%	92.6%	97.5%	95.7%	48.7%
	18-hydroxy-octadecadienoic acid	nd	nd	nd	nd	nd	nd	8.5%
	trihydroxy-octadecadienoic acid	nd	nd	nd	nd	nd	nd	8.3%
	octadecane-1.18 dioic acid	nd	nd	nd	nd	nd	nd	12.4%
	octadecene-1.18 dioic acid	nd	nd	nd	nd	nd	nd	10.3%
Total phenolic content		3.2	4.7	3.5	5.1	0.2	3.3	0.4

Total phenolic content was determined by colorimetric Folin-Ciocalteu and expressed in mg of equivalent gallic acid (for tomato) and catechin (for apple) per g of cutin monomer extracts. nd, not detected. Relative ratio of the cutin monomers determined by GC-FID.

### Effect of cutin monomer extracts on targeted apple defenses

**Figure 2:**
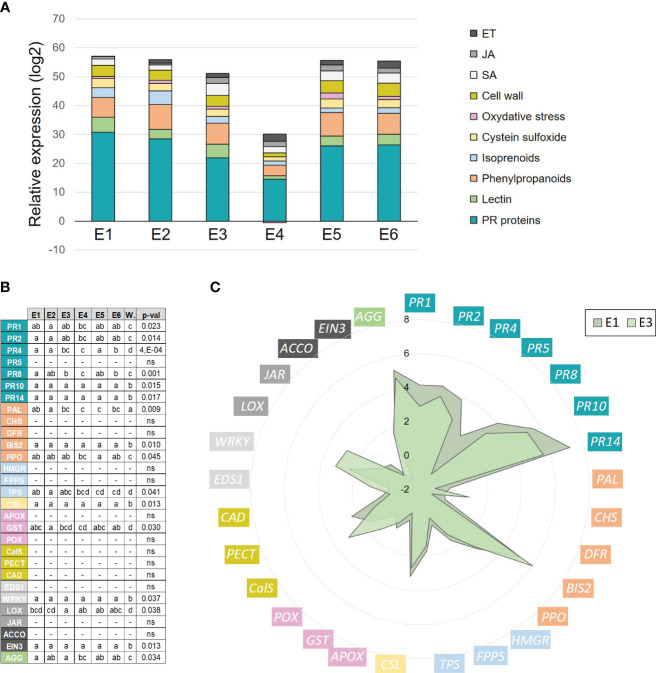
Defense-eliciting activity of crude tomato cutin monomer extracts (E1 to E6, 0.5 g/l) on apple seedlings. **(A)** Cumulative histograms of log_2_ ratios (extracts *vs.* water) obtained for 29 defense genes (grouped by defense categories) in leaves, 3 days after the second treatment. Mean values obtained from 4 biological replicates in 2 independent experiments (n = 4). **(B)** Statistical analysis of gene expression data. Letters indicate statistical classes (Kruskal-Wallis and LSD, P < 0.05). **(C)** Radar graph detailing the log_2_ ratio (extract vs. water) obtained for each gene with extracts E1 and E3. ACCO, 1-aminocyclopropane-1-carboxylic acid oxidase; AGG, agglutinin; APOX, ascorbate peroxidase; BIS2, biphenyl synthase 2; CalS, callose synthase; CHS, chalcone synthase; CAD, cinnamyl alcohol dehydrogenase; CSL, cysteine sulfoxide lyase; DFR, dihydroflavonol reductase; EIN3, ethylene insensitive 3; EDS1, enhanced disease susceptibility 1; FPPS, farnesyl pyrophosphate synthase; GST, glutathione-S-transferase; HMGR, hydroxymethyl glutarate-CoA reductase; JAR, jasmonate resistant 1; LOX, 13-lipoxygenase; PAL, phenylalanine ammonia-lyase; PR1-2-4-5-8-10-14, pathogenesis-related proteins; PECT, pectin methyl esterase; POX, peroxidase; PPO, polyphenol oxidase; TPS, terpene synthase; WRKY, WRKY transcription factor 53; W, water control.

The different tomato crude extracts obtained were evaluated in their ability to up-regulate a panel of defense genes in apple seedlings by RT-qPCR. Plants were treated twice with the different extracts (0.5 g/l) or water and the defense status of the youngest expanded leaf was assessed 3 days after the last treatment (**Supplementary Figure S1**). Overall, the different extracts induced the same defense pathways in comparison to the water control, especially the pathogenesis-related (PR), phenylpropanoid, lectin and cysteine sulfoxide genes (**Figures 2A, B**). Several observations can be made, i) the hydroxy fatty acids extracted with the alkaline-ethanol process could induce a stronger effect than those obtained with water (E3 > E4), ii) elicitation was observed with fractions extracted either from dewaxed (E1, E2, E5, E6) or non-dewaxed (E3, E4) tomato pomaces and iii) the decrease in phenolic content of the cutin monomer extract has no impact on its eliciting activity (E5 = E6). These results indicate that plant elicitation requires the cutin monomers.

Extracts E1 and E3 were chosen for further analyses because they differed only in wax presence (same batch with the best extraction yield, same depolymerization process with EtOH, similar total phenolic content). The detailed expression profiles of the defense genes obtained with these two extracts are given in **Figure 2C**. They both particularly (log_2_ ratios > 3) and significantly (P < 0.05, **Figure 2B**) induced several PR genes (*PR1*-*2*-*4*-*8*-*10*-*14*), *BIS2* (phenylpropanoid), *CSL* (cysteine sulfoxide), *WRKY53* (salicylic acid response) and *AGG* (lectin), and to a lesser extent the genes *PPO* (phenylpropanoids), *TPS* (terpenoids), *GST* (oxidative stress), *LOX* (jasmonic acid biosynthesis) and *EIN3* (ethylene response).

### Formulation and research of the optimal dose

**Figure 3:**
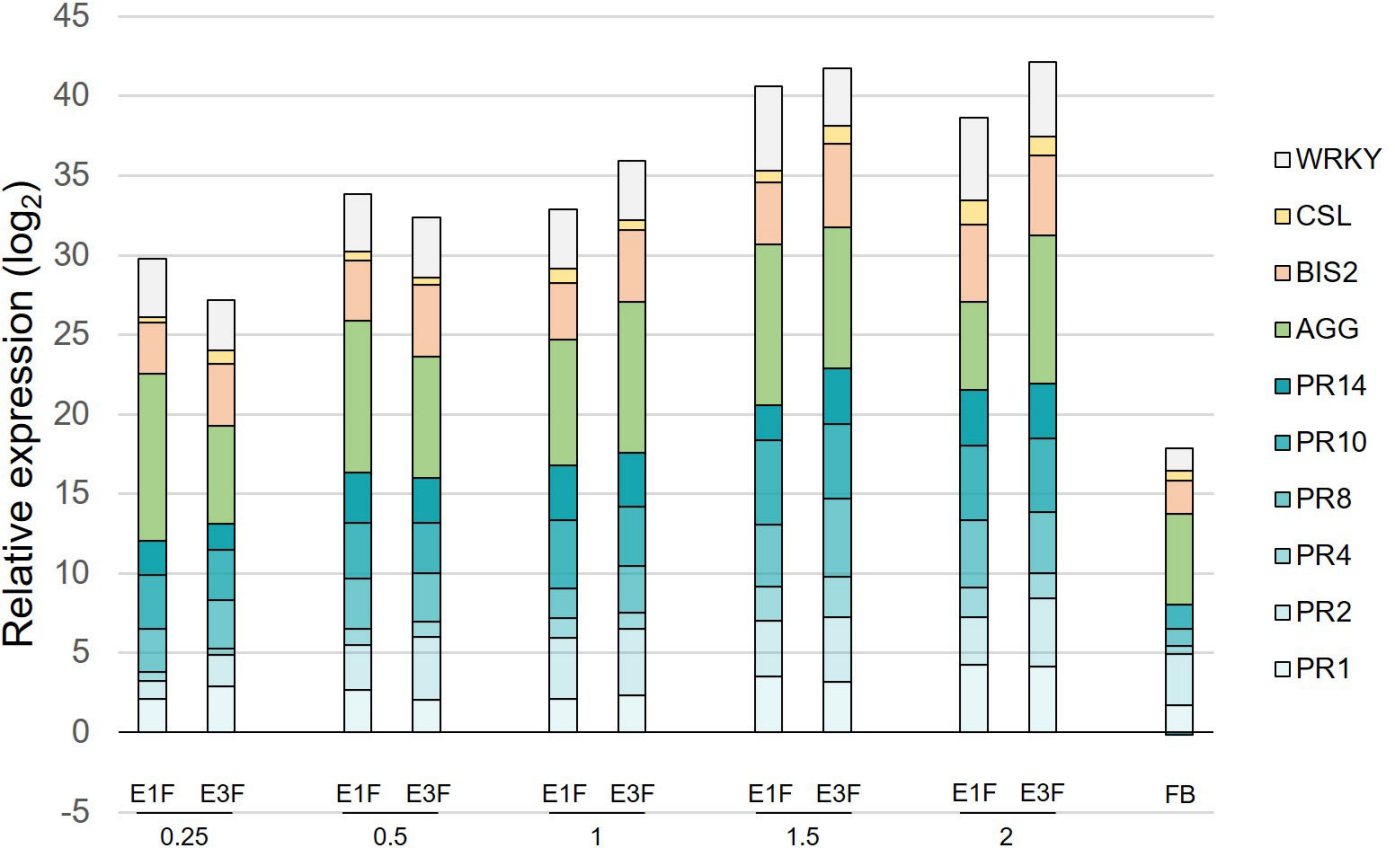
Dose-response to the formulated tomato cutin monomer extracts E1F and E3F. Cumulative histograms showing log_2_ ratios (extracts or FB vs. water) obtained for 10 defense genes in apple leaves, 3 days after the second treatment with the different doses of extracts (ranging from 0.25 to 2 g/l). Mean values obtained from 4 biological replicates in 2 independent experiments (n = 4). AGG, agglutinin; BIS2, biphenyl synthase 2; CSL, cysteine sulfoxide lyase; PR1-2-4-8-10-14, pathogenesis-related proteins; WRKY, WRKY transcription factor 53; FB, formulation blank.

Crude extracts E1 and E3 were formulated (extracts E1F and E3F) and the corresponding stock solution was diluted to evaluate the ability of increasing doses (0.25 to 2 g/l) of cutin monomer extracts to induce defense genes. The extracts were applied on apple seedlings with the same experimental design (**Supplementary Figure S1**) and the ten most reactive genes among the panel previously studied (*PR1*-*2*-*4*-*8*-*10*-*14*, *BIS2*, *CSL*, *WRKY* and *AGG*) were chosen for defense analysis. As the effect of the formulation cannot be ruled out, the formulation blank (FB) was also tested at the highest concentration to assess its effect on apple defenses (**Figure 3**). A higher elicitation (2.5-fold) was clearly observed in the presence of cutin monomers (**Figure 3**), in agreement with the elicitation observed when cutin hydroxy fatty acids were dispersed in water without formulation (**Figure 2**). A clear dose effect was observed with an induction plateau reached at 1.5 g/l of cutin monomers (at cumulative log_2_ ratios around 41). As the presence of wax did not obviously modify the eliciting activity of the extracts, the undewaxed extract E3F at the optimal dose of 1.5 g/l was chosen for further analyses.

### Comparison between tomato and apple cutin monomer extracts

**Figure 4:**
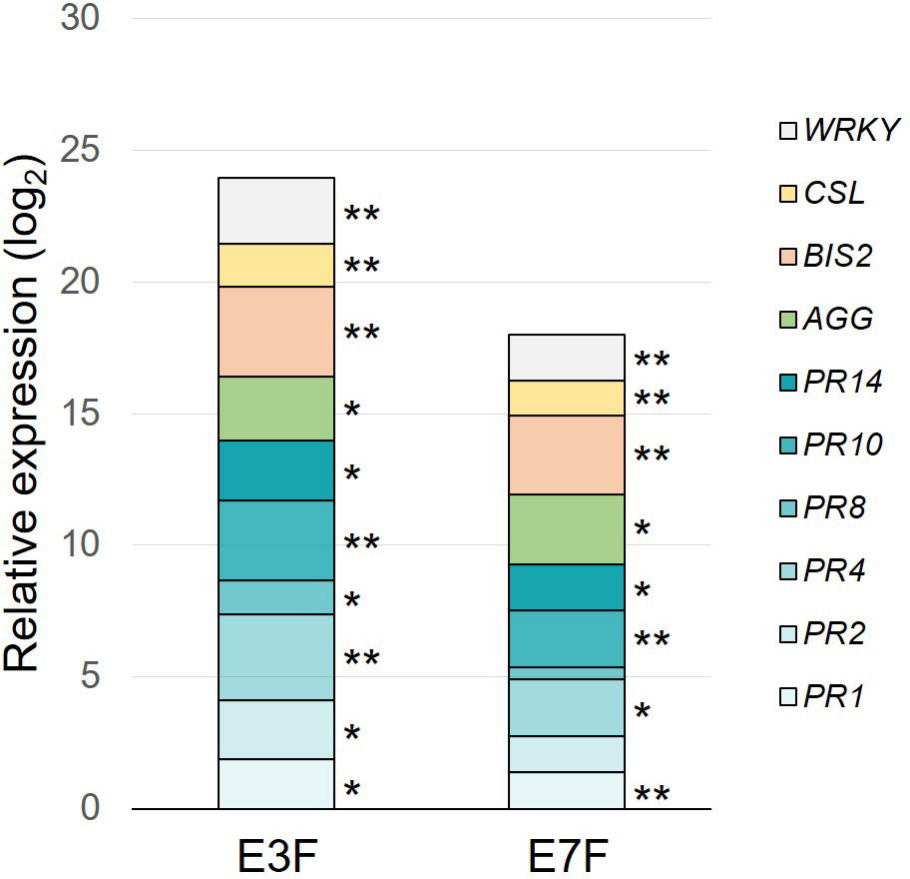
Defense-eliciting activity of the formulated tomato (E3F) and apple (E7F) cutin monomer extracts (1.5 g/l). Cumulative histograms showing log_2_ ratios (extracts *vs.* water) obtained for 10 defense genes in apple leaves, 3 days after the second treatment. Mean values obtained from 6 biological replicates in 3 independent experiments (n = 6) with P-values of LSD test (* P < 0.05 and ** P < 0.01) between water and other groups. AGG, agglutinin; BIS2, biphenyl synthase 2; CSL, cysteine sulfoxide lyase; PR1-2-4-8-10-14, pathogenesis-related proteins; WRKY, WRKY transcription factor 53; FB, formulation blank.

Extract E3F was first compared to the apple cutin monomer extract E7F (formulated at the same dose of 1.5 g/l) for its ability to induce the same 10 selected defense genes in apple seedlings (**Figure 4**). Relative to the water control, both extracts were able to induce apple defense gene expression. However, tomato extract E3F significantly induced the expression of all monitored genes whereas apple extract E7F failed to significantly induce *PR2* and *PR8* genes. Moreover, most of the genes had higher expression following E3F treatment than E7F one.

### Modulation of apple leaf transcriptome by tomato cutin monomer extract E3F

**Figure 5:**
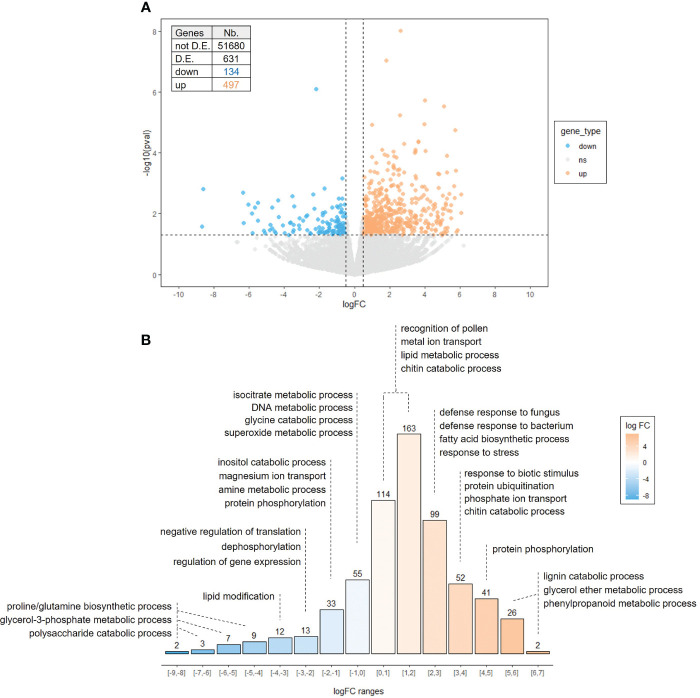
Apple leaf transcriptome response to the formulated tomato cutin monomer extract E3F (1.5 g/l). **(A)** Volcano plot representing gene expression modulation 3 days after the second treatment. Each scattered point represents a single gene: the x coordinates correspond to the log fold change (logFC) of the expression ratio between E3F- and FB-treated plants; the y coordinates correspond to –log_10_ of the p-value (n = 3 samples per treatment from 3 independent experiments). **(B)** Distribution of the number of genes by logFC ranges with main enriched Gene Ontology (GO) categories of biological process. D.E, differentially expressed.

To gain insight the mode of action of cutin monomers, and bearing in mind the slight effect of FB on plant defenses, we investigated global gene expression profiling in E3F- and FB-treated apple leaves 3 days after the second treatment (**Supplementary Figure S1**) using RNAseq analysis, to identify differentially expressed genes (DEG) with log_2_ fold changes |logFC| ≥ 0.5 and p-values ≤ 0.05. Using these criteria, 631 genes (over nearly 52,000 genes) were differentially expressed in response to E3F treatment, including 497 up-regulated and 134 down-regulated genes (**Figure 5A**). Therefore, the number of induced genes was about 4 times higher than repressed genes, with higher levels of significance achieved for several of them.

A gene ontology (GO) analysis was performed to highlight biological processes assigned to up- and down-regulated genes, and the main enriched GO terms are shown in **Figure 5B** (see **Supplementary Table S1** for a list of DEG and complete GO analysis). Down-regulated genes are rather associated with primary functions, with enriched GO terms related to amino acid (“proline and glutamine biosynthetic process”, “glycine catabolic process” and “amine metabolic process”) and carbohydrate (“polysaccharide catabolic process”, “glycerol-3-phosphate metabolic process” and “inositol catabolic process”) metabolic processes, DNA metabolic process and negative regulation of translation.

Regarding up-regulated genes, about 25% of them had high to very high expression logratios (between 3-7, **Figure 5B**). The up-regulated genes are associated with processes involved in plant immunity (GO “response to stress”, “defense response”, “response to biotic stimulus”, “chitin catabolic process”, “lignin catabolic process” and “protein phosphorylation”) including genes encoding peroxidase, basic endochitinase, laccase, pathogenesis-related proteins, disease resistance proteins and receptor protein kinases. Regardless of the GO terms enrichment, a high proportion of up-regulated genes is involved in defense mechanisms such as numerous R genes [including 46 *RLK* (receptor-like protein kinase) and 8 *RLP* (receptor-like protein)] and several JA biosynthesis-related genes [three *LOX* (13S-lipoxygenase)], transcription factor genes [*MYC4*, *ERF114*, *TIFY3B-6B-10A-10B-10C* and *WRKY6-24-40-41-48-70*], PR-proteins genes [one *PR1* (sterol binding), six *PR2* (endo-1,3-β-glucanase), one *PR4* (endochitinase type I, II), one *PR5* (thaumatin-like protein) and *PR8* (endochitinase type III) and eight *PR10* (ribonuclease like)], oxidative stress genes [six *GST* (glutathione S-transferase) and six *POX* (peroxidase)], secondary metabolism genes [two *PPO* (polyphenol-oxidase), one *BIS* (biphenyl synthase), one *NMD* (monoterpene synthase), two *TPS* (terpene synthase) and eleven *CYP450* (cytochrome P450)] (**Supplementary Table S1**). Finally, in addition to “lignin catabolism” induction [one *LAC15* (Laccase) and one *MYB15*], genes involved in cell wall remodeling [one *PAE* (pectin acetyl esterase), one *RhaT* (rhamnosyltransferase) and three *PECT* (pectin esterase inhibitor)] and cuticle biosynthesis [two *CER* (eceriferum), two *KCS* (ketoacyl-CoA synthase), two *LTP* (lipid transfer protein), four *ABCG* (ABC transporter) and six *GDSL* (GDSL esterase/lipase)] were also significantly up-regulated after the cutin monomers treatment. Interestingly, the 10 targeted genes previously observed as induced by RT-qPCR were also found up-regulated in the RNAseq analysis, albeit with a lack of significance for some of them (*PR2*, *PR14*, *BIS2*, *CSL* and *WRKY*, **Supplementary Figure S2**).

### Control of apple scab by tomato cutin monomer extracts and *in vitro* antifungal activity

**Figure 6:**
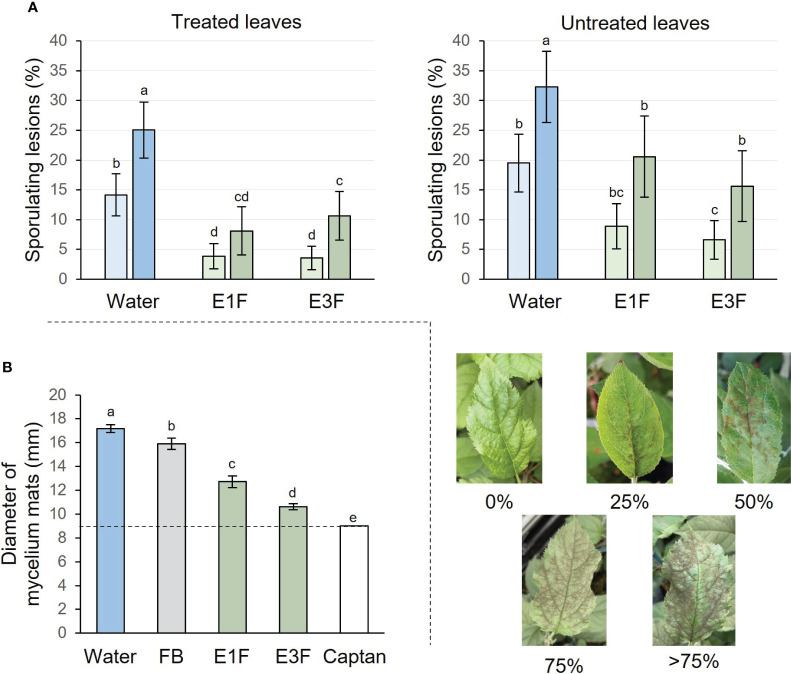
Activities of the formulated tomato cutin monomer extracts E1F and E3F (1.5 g/l) on *V. inaequalis*. **(A)** Protection tests with the extracts applied as two preventive treatments. Sporulating lesions recorded on treated or untreated apple leaves 2 (bright colors) and 3 (bright colors) weeks post-inoculation. Mean values ± 95% confidence intervals obtained from 90 plants in 3 independent experiments (n = 90). Letters indicate statistical classes (Kruskal-Wallis and LSD, P < 0.05). The pictures show different levels of disease severity on apple seedling leaves **(B)** *In vitro* biocidal effect (mycelium growth of *V. inaequalis).* Mycelium mats (diameter) measured 12 days after transferring inoculum plugs in Petri dishes containing solid medium supplemented either with E1F, E3F, FB, water or captan. Three Petri dishes with 3 plugs per condition and per experiment in at least 2 independent experiments (18 ≤ n ≤ 27). The initial diameter of the plugs is represented by the dashed line (9 mm). FB, formulation blank.

To evaluate the potential of tomato cutin monomer extracts for controlling the major disease apple scab, apple seedlings were treated twice with E1F, E3F or water and inoculated with *V. inaequalis* 2 days after the second treatment (**Supplementary Figure S1**). The symptoms development was recorded 2 and 3 weeks post-inoculation on treated and untreated leaves. Results showed that extracts E1F and E3F exhibited a similar strong protective effect against apple scab on the treated leaf, significantly reducing sporulating lesions 2 (73 or 75%) and 3 (68 or 57%) weeks post-inoculation, compared to the water treatment (**Figure 6A**). The extracts also conferred resistance to the untreated leaf with a significant reduction in disease symptoms 2 (54 or 66%) and 3 (36 or 52%) weeks after inoculation (**Figure 6A**).

Given the significant protective effect of cutin monomer extracts, it was of interest to know whether or not defense elicitation was associated with an antifungal effect. E1F and E3F were therefore evaluated *in vitro* for their effect on the mycelial growth of *V. inaequalis* and compared to negative (water and FB) and positive (captan) controls (**Figure 6B**). FB had a slight but significant effect, reducing mycelial growth by about 15% in comparison to water control. E3F was able to significantly decrease fungal mycelial growth by about 76% compared to FB, almost as much as the fully effective captan biocide control. Interestingly, the antifungal activity of E1F (wax-free extract) was lower than that of E3F with about 46% of mycelial growth inhibition compared to FB.

## Discussion

### Industrial pomaces are sources of active molecules for biotic stress management

Our present data evidenced that cutin hydroxy fatty acids extracted from tomato pomaces, highly rich in 9(10)-16-hydroxy hexadecanoic acids, are efficient to induce apple defense response, reduce the development of the fungus *V. inaequalis in vitro* and control apple scab on plants. Moreover, we used a simple and fully bio-based formulation enabling a stable dispersion of these monomers in water, taking advantage of the surface-active properties of the cutin monomers (Fameau et al., 2013). Neither the minor co-extracted phenolics compounds nor waxes had a significant impact on defense elicitation, but the presence of waxes seems to enhance their direct antifungal action. Although not as deeply studied as tomato extracts, the extract from apple pomaces seemed to share eliciting activities, which further increases the potential sourcing for the production of cutin monomer extracts. Moreover, the amount of cuticle extracts needed for plant protection is consistent with the amount of cutin-enriched industrial pomaces produced each year. Indeed, the estimated annual worldwide pomaces (including peel, seed, and pulp) is 4.10^6^ tons/year for apples (Costa et al., 2022) and 6 to 9.10^6^ tons/year for tomatoes (Lu et al., 2019), respectively. According to the peel ratio in these pomaces (Escórcio et al., 2022; Mellinas et al., 2022) and our extraction yield, this represents a potential source of at least 500-600.10^3^ tons/year of hydroxy fatty acids extract. Altogether, these results are in line with a sustainable bioeconomy and the development of a viable agronomic bio-sourced strategy against fungal infection.

### Cutin monomer extracts trigger several plant immune mechanisms

**Figure 7:**
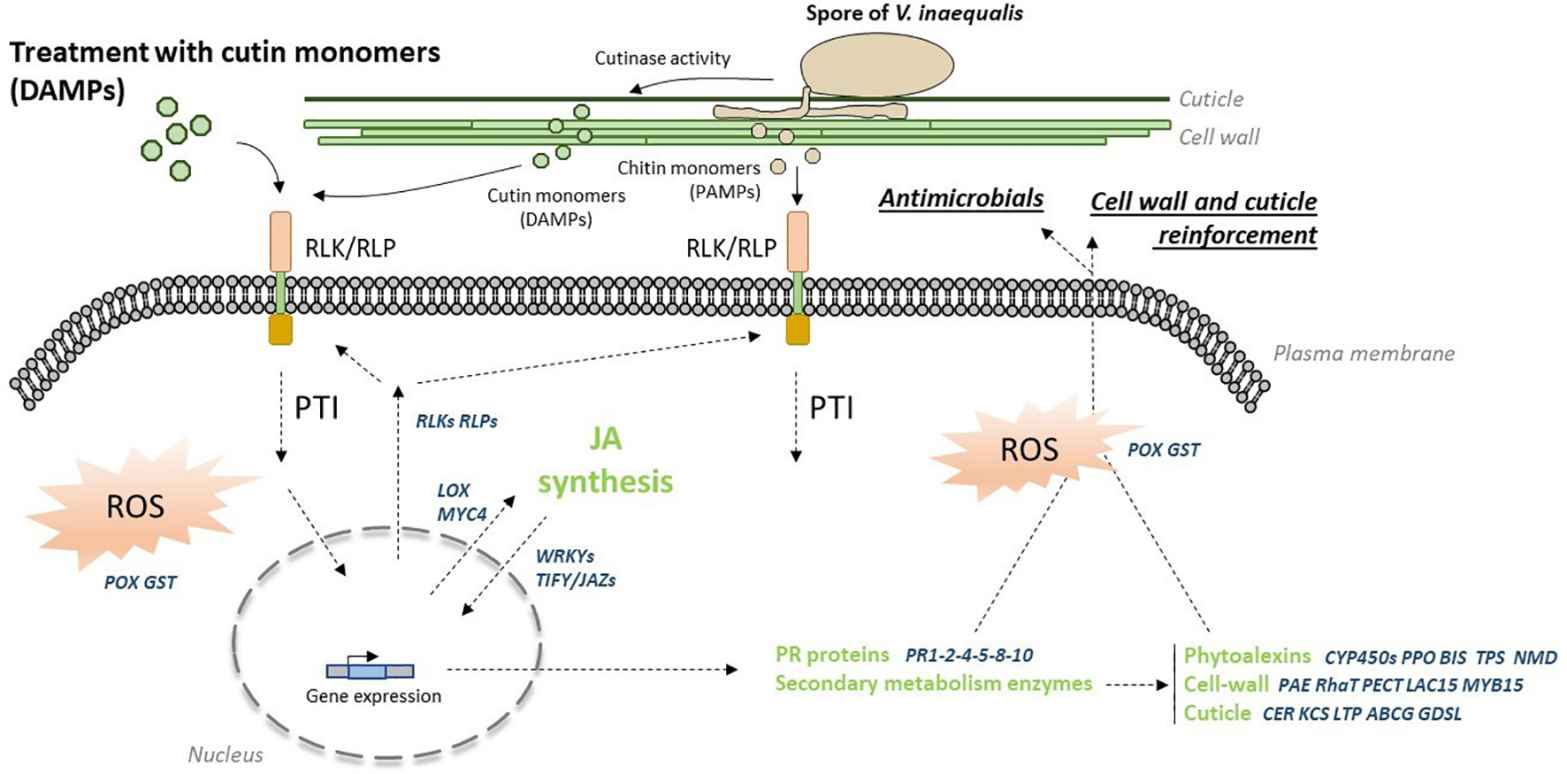
Hypothetical immune response to cutin monomers treatment. Cutin monomers are probably recognized by RLK/RLP receptors whose expression is notably up-regulated after recognition (these receptors could recognize cutin monomers released by the cutinase activity of *Venturia inaequalis* and also chitin monomers from the fungus). Recognition of these motifs triggers a PTI-like response, including the initiation of an oxidative burst. JA synthesis is induced, triggering the expression of defense-related genes. The biosynthesis of antimicrobial PR proteins and phytoalexins is then initiated, along with that of the cell wall, cuticle and wax components, leading to the establishment of physical and chemical barriers against the fungus. ABCG, ABC transporter G; BIS, biphenyl synthase; CER, eceriferum; CYP450s, cytochrome P450 proteins; DAMPs/PAMPs, damage/pathogen-associated molecular patterns; GDSL, GDSL esterase/lipase; GST, glutathione-S-transferase; KCS, ketoacyl-CoA synthase; LAC15, laccase 15; LOX, 13-lipoxygenase; LTP, lipid transfer protein; MYB15, MYB transcription factor 15; MYC4, MYC transcription factor 4; NMD, monoterpene synthase; PAE, pectin acetyl esterase; PECT, pectin esterase inhibitor; POX, peroxidase; PPO, polyphenol oxidase; PR1-2-4-8-10-14, pathogenesis-related proteins; PTI, PAMP-triggered immunity; RhaT, rhamnosyltransferase; RLK/RLP, receptor-like protein kinase/receptor-like protein; ROS, reactive oxygen species; TIFY/JAZs, jasmonate ZIM-domain protein; TPS, terpene synthase; WRKYs, WRKY transcription factors.

RNAseq analysis highlights different defense mechanisms induced by hydroxy fatty acids. A schematic representation of the immune response triggered by cutin monomer extracts is shown in **Figure 7**. The large number of *RLP* and *RLK* genes induced suggests the establishment of an immune response in apple. These receptors are involved in many biological processes with some known to be involved in the recognition of PAMPs (e.g. flagellin, lipopolysaccharides, fungal chitin) and DAMPs (e.g. oligogalacturonides) to trigger PAMP-triggered immunity (PTI) (Yang et al., 2012). Induction of these receptors could i) amplify the response to a second treatment with cutin monomers, ii) enable recognition of monomers derived from cutin hydrolysis by the cutinase activity of *V. inaequalis* (Köller, 1989) and/or iii) chitin monomers from the fungus (Rocafort et al., 2023). However, these receptors are not well characterized in apple and their ability to recognize cutin (DAMP) or chitin (PAMP) monomers has yet to be demonstrated. RNAseq data provide evidence that treatment activates the jasmonic acid (JA) signaling pathway, through the overexpression of i) three *LOX* genes (13S-lipoxygenase), the initial enzymes of the JA biosynthesis pathway (Bell et al., 1995) and ii) the transcription factor MYC4, acting together with MYC2 and MYC3 to activate JA responses (Zhang et al., 2020). Among the targets of MYC factors are the *TIFY/JAZ* genes (jasmonate-zim-domain proteins) encoding repressors of MYC2 (Fonseca et al., 2009) and up-regulated in our experiments. This result is consistent with the transcriptional negative feedback described in the JA signaling pathway to modulate JA response (Fonseca et al., 2009). Several WRKY transcription factors, known as key regulators in plant immune response to various biotic stresses (Wani et al., 2021), were also up-regulated by the cutin monomer treatment. Described as major players in hormone signaling networks, their overexpression may also suggest the involvement of other phytohormones in the plant response to cutin monomers.

A high number of PR genes (18 genes encoding PR1, PR2, PR4, PR5, PR8 or PR10) were induced after cutin monomers treatment, indicating the synthesis of defense end-players. Indeed, PR proteins are inducible defense-related proteins upon infection with various pathogens and many of them exhibit antimicrobial properties (Van Loon et al., 2006). Four PR-proteins evidenced in our study, i.e. the β-1,3-endoglucanase PR2, the thaumatin/osmotin-like PR5 and the endochitinases PR4 and PR8, might be involved in the control of *V. inaequalis* on apple seedlings after treatment through plasma membrane or cell wall weakening (Van Loon et al., 2006). The sterol-binding activity of PR1 rather targets sterol-auxotroph pathogens such as some oomycetes (Gamir et al., 2017), unlike *V. inaequalis* which is sterol-autotroph. The ribonuclease-like protein PR10, well-known as the major allergen Mal d1/Bet V1 of apple fruits, has rather an antiviral action (Park et al., 2004).

Downstream defense molecules with antimicrobial activity can also be derived from the secondary metabolism. The RNAseq analysis highlighted several interesting genes in that matter. Transcript accumulation of two *PPO* genes (polyphenol oxidase) after treatment suggests an increased ability to produce oxidation products from phenolics. Gaucher et al. (2013) previously showed that dihydrochalcones, the major flavonoid subgroup in apple leaves, can lead to highly bactericidal compounds against the bacterial apple pathogen *Erwinia amylovora* after deglucosylation followed by oxidation through PPO action. Their fungicidal activity against *V. inaequalis* remains however to be tested. The induction of *BIS* (biphenyl synthase) also suggests the synthesis of biphenyls and dibenzofurans whose inhibitory activity has already been described on the growth of *E. amylovora* (Chizzali et al., 2012a) and the spore germination and growth of *V. inaequalis* (Hrazdina et al., 1997; Sarkate et al., 2018). The presence of *CYP450* in the list of up-regulated genes by treatment also points in this direction. Cytochrome P450s represent a large enzyme family involved in NADPH/O_2_-dependent hydroxylation reactions leading in particular to secondary metabolites and phytohormones (Pandian et al., 2020). Interestingly, the *CYP736A12* up-regulated in our study belongs to a family whose members encode a biphenyl 4-hydroxylase leading to the formation of aucuparin, a major biphenyl compound in apple (Sircar et al., 2015). The overexpression of two *TPS* genes (terpene synthase) also suggests the emission of volatile compounds in response to treatment. Such induction of *TPS* genes has already been described in apple leaves treated with the elicitor acibenzolar-S-methyl, accompanied by emission of the sesquiterpene (E,E)-α-farnesene exhibiting a significant repellent effect against the rosy apple aphid *Dysaphis plantaginea* (Warneys et al., 2018). Thus, the modulation of genes belonging to different pathways of secondary metabolism by cutin monomer treatment could indicate that the induced response affects a larger spectrum of apple pests than just *V. inaequalis*.

There is evidence that the cell wall and the cuticle undergo dynamic remodeling in response to pathogen infection, allowing maintenance/reinforcement of their components as well as the release of potent elicitors (Voisin et al., 2009; Wan et al., 2021). Our present RNAseq data are in accordance with these results, since pectin (*PAE*, *PECT*, *RaHT* genes) and lignin (*LAC15* and *MYB15* genes) seemed to undergo dynamic changes after cutin monomer treatments, as well as cuticle polymers (*CER*, *KCS*, *GDSL*, *LTP* and *ABCG* genes). Interestingly, the connection between SAR (*PR1* expression) and wax biosynthesis was also identified in *cer6* and *cer2 Arabidopsis thaliana* mutants (Garbay et al., 2007). More recently, wax crystal accumulation was associated with resistance to *Botryosphaeria dothidea* in apple (Zhang et al., 2019). Altogether, our data indicate that remodeling of these polymers could be part of the PTI response triggered by the cutin monomer treatment. These results are in full agreement with the crosstalk of cuticle, plant cell wall and hormone signaling pathways, that has been suggested in plant disease resistance (Ziv et al., 2018).

Reactive Oxygen species (ROS) can reach toxic levels acting directly as antimicrobials or participate in various events occurring during the plant immune responses such as programmed cell death, signal transduction pathways and cell wall reinforcement (Mansoor et al., 2022). Our data suggest ROS accumulation in apple leaf treated by cutin monomers through the induction of several *POX* (peroxidase) and *GST* (glutathione-S-transferase) genes. This result is consistent with the H_2_O_2_ production observed in cucumber hypocotyls treated with monomers from hydrolysates of cucumber, apple and tomato cutins (Fauth et al., 1998). It has also been reported that different *Arabidopsis* mutants affected in their cuticular structure spontaneously accumulate ROS (Serrano et al., 2014).

### Cutin monomer extracts can also directly impact the pathogen

The protection efficacy of cutin monomers against apple scab was probably not exclusively linked to the triggering of a defense response in apple, but also to the biocidal effect of these compounds observed *in vitro*. Indeed, we observed that the cutin monomer extracts could inhibit the mycelium growth of *V. inaequalis.* The antimicrobial activity of fatty acids has been the subject of numerous studies and controversial reports have been published leading to the conclusion that their antimicrobial activities are related to specific structural features (e.g. chain length, unsaturation degree, position of hydroxyl groups) and targeted pathogens (Pohl et al., 2011; Guimarães and Venâncio, 2022). Whatever the structure of long-chain fatty acids, the antimicrobial activity is mainly related to their solubility in aqueous solution. Indeed, only fatty acid salts are soluble in water where they are dispersed as micelles above their CMC (critical micellar concentration) while the protonated form is water-insoluble. The molecular-micelle equilibrium facilitates the molecular transfer of fatty acids in the lipid bilayer membranes where they can disrupt their integrity and induce cell death, a mechanism generally admitted to explain their antimicrobial properties (Arellano et al., 2023). This could explain in previous studies why cutin monomers can confer disease resistance without displaying any antifungal activity, since they were dispersed in their protonated insoluble form (Schweizer et al., 1994; Schweizer et al., 1996). In our study, the cutin monomers were dispersed in water as their salts, leading to the formation of small vesicles and not micelles (Fameau et al., 2013). As recently suggested (Arellano et al., 2023), these vesicles could easily fusion with the cell membrane of the microbial pathogen leading to the full and rapid transfer of the fatty acids into the membrane of the targeted pathogen. Furthermore, we observed that the presence of residual tomato waxes enhances the biocide activity of the formulated cutin monomer extracts. Tomato waxes are mainly composed of saturated long-chain alkanes and triterpenoids, i.e. amyrins (Girard et al., 2012). Many fruit wax triterpenoids display antimicrobial properties (Szakiel et al., 2012) and especially β-amyrin displays both antifungal and antibacterial activities (Kwun et al., 2021; Han and Lee, 2022). Like cutin in cuticles, the cutin monomer extracts should facilitate the solubilization of the hydrophobic tomato wax amyrin and its transfer and apoptotic action on microbial cells.

## Conclusion

The development of bio-based strategies for plant protection is imperative to meet the requirements of a sustainable agriculture. The first steps towards this objective were achieved in this work, using abundant sources of agricultural wastes to produce monomer extracts by an effective biorefinery process simple-to-implement at an industrial scale. For agronomical use in plant protection, we propose a stable dispersion of these cutin monomers in water, fostering their dual biological activities, i.e. an eliciting activity via jasmonate, secondary metabolism and ROS pathways as well as an antifungal action. The next steps will be to confirm the performance of the formulated preparation in orchard and to explore other pathosystems to reveal the full potential of cutin monomers in the management of biotic stresses.

## Data availability statement

The datasets presented in this study can be found in online repositories. The names of the repository/repositories and accession number(s) can be found below: NCBI GSE245554.

## Author contributions

MG: Conceptualization, Data curation, Investigation, Methodology, Supervision, Writing – original draft. AJ: Conceptualization, Methodology, Writing – review & editing. BN: Methodology, Writing – review & editing. NV: Methodology, Writing – review & editing. CE: Funding acquisition, Project administration, Writing – review & editing. DM: Conceptualization, Methodology, Writing – review & editing. MB: Conceptualization, Supervision, Writing – review & editing. BB: Conceptualization, Funding acquisition, Investigation, Methodology, Supervision, Writing – original draft.
