# Supplementary figures and images for: Formulated hydroxy fatty acids from fruit pomaces reduce apple scab development caused by Venturia inaequalis through a dual mode of action
Source: Front Plant Sci. 2024 Jan 8;14:1322638. doi: 10.3389/fpls.2023.1322638 (PMC10800985; doi:10.3389/fpls.2023.1322638)

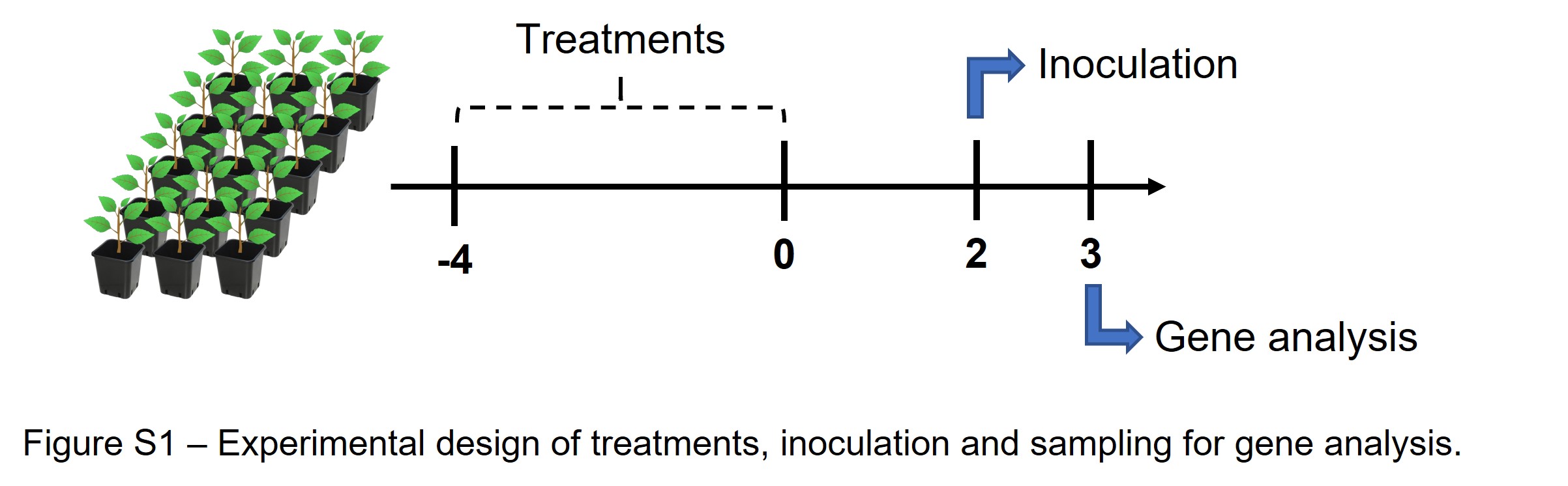

Supplement: Supplementary file 2 [file Image_1.jpeg]

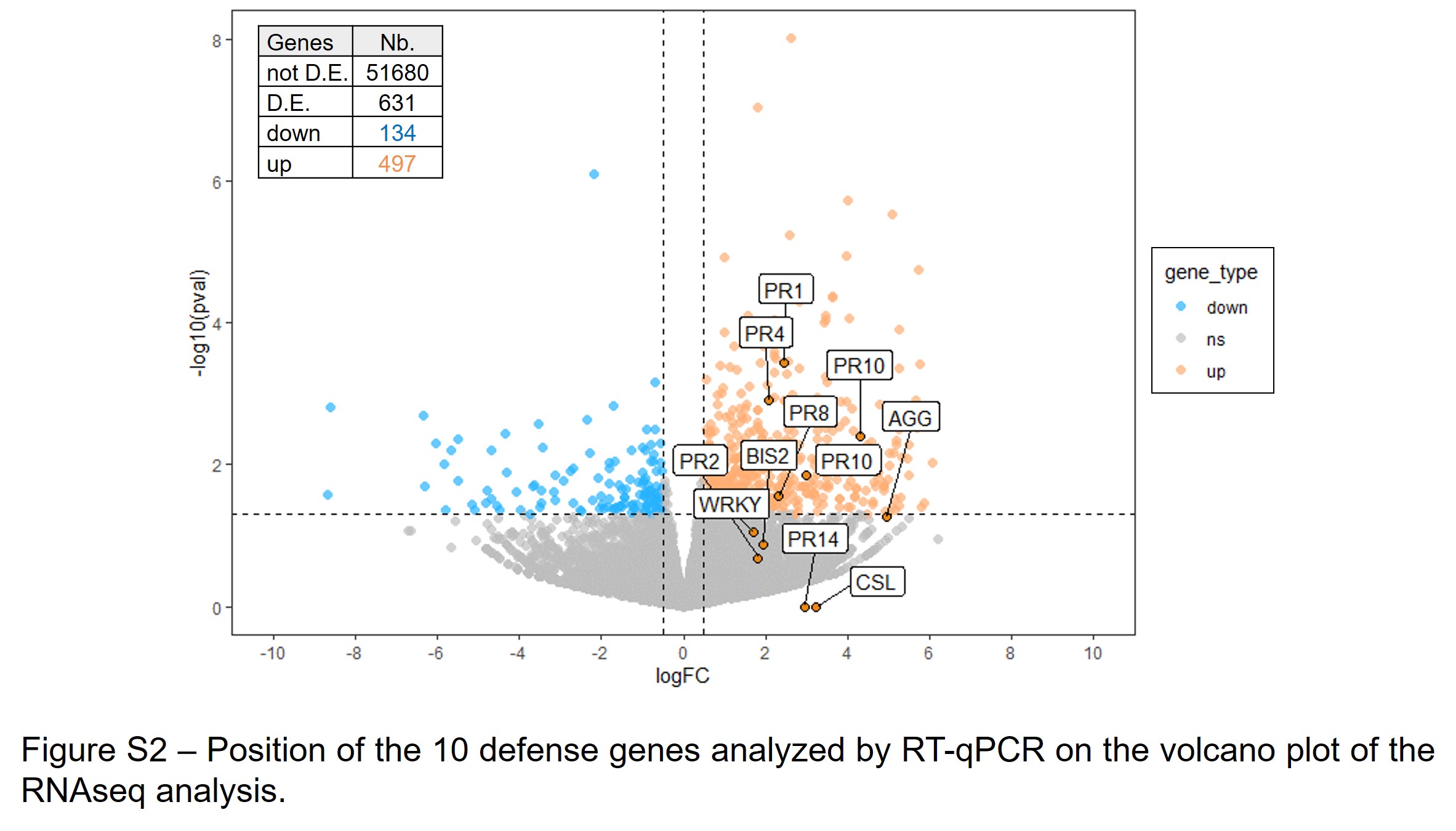

Supplement: Supplementary file 3 [file Image_2.jpeg]
